# Psychoeducational groups for relatives of patients with cognitive impairment: Effect on the psychological state of caregivers

**DOI:** 10.1192/j.eurpsy.2021.1357

**Published:** 2021-08-13

**Authors:** J. Marti Bonany, M.I. Martínez Casamitjana, C. Macias Castellví, J.R. Fortuny Olive, M.T. Campillo Sanz, G.A. Mateu Codina, R.B. Sauras Quetcuti, M. Vallve Elias, R. Sánchez González, M. Pérez Carre

**Affiliations:** Institut De Neuropsiquiatria I Addiccions, Parc de Salut Mar, Santa Coloma de Gramenet, Spain

**Keywords:** psychoeducational, group, relatives, cognitive impairment

## Abstract

**Introduction:**

The Cognitive Disorders Unit carries out sessions of Psychoeducational Groups (PG) for caregivers of patients diagnosed with cognitive impairment (CI). The aim is to educate about the disease, improve the caregiver’s self-care and learn how to take better care of the sick.

**Objectives:**

Analyze the profile of the caregivers that participate in PG and assess changes in their psychological state.

**Methods:**

Subjects: 110 caregivers of patients diagnosed with mild-moderate CI who have participated in PG. Methodology: sociodemographic data of the caregiver and patient are collected. The following scales are passed: General-Health-Questionnaire (GHQ-12), Global-Deterioration-Scale, Barthel-Index. 5 sessions of 90 minutes are carried out every fortnight. An opinion questionnaire and the GHQ-12 are administered at the end of the sessions.

**Results:**

86% of caregivers are women: 37% spouses and 55% daughters; mean age 57; 92% of patients live with the caregiver. 62% of caregivers present some kind of psychological disorder that is significantly reduced (p=0,0003) after some sessions. After PG: 65% of caregivers are able to further enjoy their daily activities 46% improve concentration capacity 42% improve sleeping and mood. Opinion Questionnaire Results: 98% of caregivers are satisfied with the activities, the topics addressed and their applicability.

**Conclusions:**

The participants in PG were mostly daughters of patients, with average age 57, and living in the same household. Participation in PG improves the information and skills of caregivers, and reduces psychological disorders by improving their mood, their ability to concentrate, their quality of sleep and enjoyment of daily activities.

